# *In Vivo* Expression Pattern of MICA and MICB and Its Relevance to Auto-Immunity and Cancer

**DOI:** 10.1371/journal.pone.0000518

**Published:** 2007-06-13

**Authors:** Stéphanie Schrambach, Marc Ardizzone, Vincent Leymarie, Jean Sibilia, Seiamak Bahram

**Affiliations:** 1 Laboratoire Central d'Immunologie, Pôle de Biologie, Hôpitaux Universitaires de Strasbourg, Strasbourg, France; 2 Service de Rhumatologie, Hôpitaux Universitaires de Strasbourg, Strasbourg, France; 3 Immunogénétique Moléculaire Humaine, Centre de Recherche d'Immunologie et d'Hématologie, Faculté de Médecine, Strasbourg, France; 4 Laboratoire d'Hématologie, Hôpitaux Universitaires de Strasbourg, Strasbourg, France; Centre de Recherche Public-Santé, Luxembourg

## Abstract

Non-conventional MHC class I MIC molecules interact not with the TCR, but with NKG2D, a C-type lectin activatory receptor present on most NK, γδ and CD8^+^ αβ T cells. While this interaction is critical in triggering/calibrating the cytotoxic activity of these cells, the actual extent of its *in vivo* involvement, in man, in infection, cancer or autoimmunity, needs further assessment. The latter has gained momentum along with the reported expansion of peripheral CD4^+^CD28^−^NKG2D^+^ T cells in rheumatoid arthritis (RA). We first initiated to extend this report to a larger cohort of not only RA patients, but also those affected by systemic lupus erythematosus (SLE) and Sjögren's syndrome (SS). In RA and SS, this initial observation was further tested in target tissues: the joint and the salivary glands, respectively. In conclusion and despite occasional and indiscriminate expansion of the previously incriminated T cell subpopulation, no correlation could be observed between the CD4^+^CD28^−^NKG2D^+^ and auto-immunity. Moreover, *in situ*, the presence of NKG2D matched that of CD8^+^, but not that of CD4^+^ T cells. In parallel, a total body tissue scan of both *MICA* and *MICB* transcription clearly shows that despite original presumptions, and with the exception of the central nervous system, both genes are widely transcribed and therefore possibly translated and membrane-bound. Extending this analysis to a number of human tumors did not reveal a coherent pattern of expression vs. normal tissues. Collectively these data question previous assumptions, correlating a tissue-specific expression/induction of *MIC* in relevance to auto-immune or tumor processes.

## Introduction

Cytotoxic CD8^+^ and helper CD4^+^ αβ T cells recognize conventional peptide-loaded Major Histocompatibility Complex (MHC) class I and II molecules respectively [1]. Whilst the former interaction leads to the elimination of the virally infected or the tumorized target cells, the latter, helps amplify/regulate primarily the humoral immune response against exo or auto-antigens [2], [3]. Although this textbook definition of the adaptive immune response is clear, real life functional/dysfunctional implications of these interactions, in conjunction with the second, innate, actors of the immunity, is far from being simple. This is perhaps best exemplified in auto-immune reactions, where depending on the disease, the species (human or mouse in essence) and the experimental model, different components of the immune system have been sequentially put to the forefront, leading to the logical assumption that virtually all actors of the immune system are involved either in triggering and/or perpetuation of the disease [4]–[6].

Unlike the above mentioned conventional MHC-I molecules, MIC molecules [7], [8] do not interact with the TCR *per se* but do so with NKG2D, a C-type lectin activatory receptor expressed on NK, γδ and CD8^+^ αβ T cells [9], [10]. This interaction has been shown to over-ride the inhibitory signal provided by Killer Inhibitory Receptors (KIR) and/or CD94/NKG2A/B molecules which sense the presence of respectively HLA-ABC and -E on target cells. While on T cells, NKG2D functions as a co-stimulatory molecule [11], it may activate NK cells as well as IL-15-activated intraepithelial T lymphocytes in a TCR independent fashion [12]. MIC-NKG2D engagement is believed to be consequently critical in eliminating infected cells and/or tumours [13], [14]. Whether this interaction is also relevant in autoimmunity is a matter of more recent emphasis [15].

Rheumatoid arthritis (RA) is an archetypical auto-immune disease and as such resumes our present knowledge and challenges regarding autoimmunity [16]. A vast body of work has yielded to numerous hypotheses regarding the initiation and perpetuation of the disease. Throughout time, T cells or B cells have been recognized as the primary actors of the disease. The putative tissue-specific or systemic auto-antigen(s) in man remains elusive, in contrast to several natural or transgenic/gene targeted animal models where several self antigens have been recognized to able to initiate the disease [17]. One of the recent putative actors in RA pathogenesis has been forwarded as a discrete population of CD4^+^ T cells which lack the co-stimulatory molecule CD28 and have been reported to be expanded in RA [18], [19]. More recent investigations reported that these T cells express, instead of CD28, the above described NKG2D [15]. The following work was initiated on this assumption and aimed to first replicate these initial data in a larger, better defined, cohort of RA patients. It was further extended to two other auto-immune diseases: systemic lupus erythematosus (SLE) and Sjögren's syndrome (SS). In RA and SS afflicted patients it was also possible to probe *MIC* expression level in target tissues, both at the messenger RNA and the protein level. Moreover in peripheral blood besides measuring the presence of CD4^+^NKG2D^+^ cells per se, we also set to analyze the TCR β repertoire of these cells in certain individuals (cf. *infra*). Finally, and for the first time, a comprehensive organ/tissue scan allowed to uncover the fact that in contrast to what was initially assumed and has since entered textbooks [20], and with the exception of the central nervous system, *MICA* and *MICB* were transcribed in virtually every tissue/organ examined. The further extension of this analysis to tumorized samples helped give a better grasp of *MIC* transcription in both health and disease.

## Materials and Methods

### Patients and controls

25 healthy volunteers as well as 75 patients suffering from RA (n = 35; 1988 American College of Rheumatology criteria)[21], SLE (n = 15; 1997 revised American College of Rheumatology criteria)[22] and SS (n = 25; 2002 American and European criteria)[23] were included in this study (Table 1). All samples were obtained from volunteers attending the Rheumatology clinic of Strasbourg University Hospitals and were collected during routine clinical (diagnostic/prognostic/therapeutic) procedures prescribed by one of the co-authors, Dr. J. Sibilia. Written informed consent was obtained from each individual in agreement with the Helsinki Declaration and French legislation, under which no approval by an ethical committee was required in this case. All patients and controls were unrelated.

**Table 1:** Characteristics of patients and healthy controls.

	Controls	RA	SLE	SS
**Individuals (men/women)**	25 (3/22)	35 (5/30)	15 (3/12)	25 (1/24)
**Mean age**	37.5 (25–58)	49.7 (30–77)	38 (16–70)	56.5 (40–80)
**Disease duration (years)**	–	6.8 (1–25)	6.6 (3–16)	11.6 (2–42)
**Disease activity** *	–	4.7 (1.8–7.4)	0.5 (0–5)	64.6 (25–96.6)
**No treatment**	–	5 (14%)	3 (20%)	15 (60%)
**NSAID** **	–	8 (23%)	0	0
**Corticosteroids**	–	12 (40%)	6 (20%)	3 (12%)
**SMARD**	–	7 (20%)	6 (40%)	4 (16%)
**DCART**	–	13 (37%)	6 (40%)	3 (12%)
**ANA>320**	–	5 (14%)	14 (93%)	15 (60%)
**ENA**	–	0	7 (47%)	12 (48%)
**Anti-dsDNA**	–	0	3 (20%)	0
**RF**	–	21 (60%)	1 (7%)	6 (24%)
**Anti-CCP**	–	15 (43%)	0	0
**Joint erosions**	–	22 (63%)	0	0

* DAS (Disease Activity Score) 28 for RA, SLEDAI (Systemic Lupus Erythematosus Disease Activity Index) for SLE and mean AVS (AVS: Analogic Visual Scale) of subjective sicca syndrome, asthenia and pain for SS.

** Abbreviations: NSAID: Non-Steroidal Anti-Inflammatory Drugs; SMARD: Symptom Modifying Anti-Rheumatic Drugs; DCART: Disease Controlling Anti-Rheumatic Therapy; ANA: antinuclear antibody (indirect immunofluorescence on Hep2 cells), ENA: extractable nuclear antigens; dsDNA: double-stranded DNA, RF: Rheumatoid Factor, CCP: cyclic citrullinated peptide.

### Flow Cytometry

The following, variously conjugated (PE, FITC, PerCP, Cy5 or APC) antibodies were used in three- and -four-color flow cytometry: anti -CD3, -CD4, -CD5, -CD8, -CD16, -CD19, -CD28, -CD45, -CD45RA, CD45RO, -CD56, -CD94, -CD158a, -CD158b, -NKG2A, -TCR γδ. The T cell repertoire was analysed by the following PE and FITC conjugated anti-TCRV_β_ (1, 2, 3, 4, 5.1, 5.2, 5.3, 7.1, 7.2, 8, 9, 11, 12, 13.1, 13.2, 13.6, 14, 16, 17, 18, 20, 21.3, 22, 23) (Beckman Coulter) antibodies. Isotype-matched controls were used for each staining. NKG2D expression was analysed using PE-conjugated ON72 (Beckman Coulter) and 1D11 (BD PharMingen), either PE-conjugated or biotinylated; the latter detected by streptavidine-PC5 (BD PharMingen). Peripheral blood cells were studied either unfractionated or after Ficoll density gradient centrifugation. Mononuclear cells were also isolated by Ficoll density gradient centrifugation from articular fluids obtained from 1 RA, 3 osteoarthritis and 2 non auto-immune arthritis patients. Flow cytometry was performed on either a BD FACSCalibur™ (Becton Dickinson) or FC 500 (Beckman Coulter) systems.

### Immunohistochemistry

Synovial tissues were obtained from 1 RA elbow and 1 osteoarthritis patients at the time of knee arthroplasty. Salivary gland tissues were obtained from 4 SS and 1 healthy volunteer during rhinoplasty. For immunohistochemistry, 4 µm cryostat sections were made from synovial tissues or salivary glands and snap-frozen in liquid nitrogen. Successive sections were fixed in acetone, air dried, rehydrated in PBS, and blocked with 0.3% Hydrogen peroxide. Sections were incubated with anti: -NKG2D mAb 1D11 (BD PharMingen), -MIC mAb 6D4 (BD PharMingen), -CD4, -CD8 for 1 h at room temperature. Binding was detected using biotinylated secondary anti-IgG and streptavidin peroxidase. Sections were counterstained with Harris' hematoxylin and mounted with Glycergel.

### Northern Blotting

Human mRNA blots were purchased from Invitrogen (Invitrogen Northern Territories). The membranes were sequentially hybridized with *MICA, β-actin* and *β_2_-microglobulin (β_2_m)* cDNAs, subjected subsequently to high stringency washes (64°C, 0.1XSSC, 0.1% SDS), prior to exposure to Kodak Xomat (Eastman Kodak, Rochester, NY) for respectively 8h at −70°C, 1 h at room temperature and 4h at −70°C respectively.

### Real-time quantitative PCR

Accessory salivary gland biopsies were obtained from an independent set of 20 (19 female and one male; average age 51, median 52) patients with primary SS. Salivary glands from 5 patients undergoing orofacial surgery (for independent causes) were obtained during the procedures and served as controls while both pathology as well as patient history further eliminated any signs of auto-immunity in these control individuals. Total cellular RNA was isolated by TRIzol LS® (Invitrogen) according to manufacturer's recommendations. The quality of extracted RNA was ascertained through gel electrophoresis. 1 µg of total RNA was subjected to reverse-transcription using oligo-dT and the ImProm-II™ Reverse Transcription System (Promega), following manufacturer's recommendations. Real-time quantitative PCR was performed on an ABI PRISM 7000 using the following oligonucleotides and TaqMan® probes. The expression levels of *MICA* and *MICB* were calibrated using that of the house keeping 18S RNA detected using the following forward and reverse primers respectively 3′-CGGCTACCACATCCAAGGAA-5′, 3′-GCTGGAATTACCGCGGCT-5′ and SYBR Green. *MICA* transcripts were detected using the following forward, reverse oligonucleotides and TaqMan® probe respectively 3′-CAGCTGCAAACGCCTCATATC-5′, 3′-TGACCTATGAAACAGAGAAAATAAAAGC-5′ and 3′-ACATTTTGCAGCCTCCAACAACAATAAATAAGTG-5′ and those of *MICB* with F 3′-CACCCAGGCTGCAGTTCACT-5′, R 3′-CGGGAGTCTGAGGTACGAGAA-5′, 3′-ACCTCTGCCTCCCAGGTTCAAGCAC-5′ in the same order as above. Positive upregulation of *MIC* transcription was ascertained in HeLa cells grown untreated or heat shocked as previously described [24].

## Results and Discussion

Despite the fact that the initial “glitch” triggering auto-immune diseases in general and rheumatoid arthritis in particular, remains mostly enigmatic, decades of research have brought together several plausible scenarios whereas upon initiation, various pathways and cascades tend to more or less perpetuate an auto-reactive (T and/or B cell driven) force, eventually leading to symptomatic pathology [6], [25]–[28]. One of the latest incriminated T cell subpopulations as such, is the fraction of CD4^+^ helper T cells expressing the activating receptor NKG2D, but devoid of the co-stimulatory molecule CD28 [15]. NKG2D, a type-II trans-membrane surface homodimer consists of a C-type lectin extracellular domain, followed by a transmembrane and a short cytoplasmic tail devoid of any particular signalling function. The molecule signals indeed (at least in man) via interaction with the trans-membrane adaptor protein DAP10 (DAP10 and DAP12 in mus depending on the NKG2D isoform)[29]. NKG2D recognizes a variety of distantly related non-conventional MHC class I molecules; in man MIC [7] and RAET1 [30] (ULBP) [31] and in mouse RAE1 and H60 [9], [29]. The initial aim of this work was to advance our knowledge of the potential role of the MIC-NKG2D interaction in autoimmunity in man. In order for such to be the case, (at least) two conditions should be met: (1) firstly and ideally, organs target for autoimmunity (hence potentially all organs and/or tissues), should not express MIC at basal state, in other words, MIC should be only detected in diseased tissues or pathological states. (2) secondly that systemic and/or tissue-infiltrated autoreactive CD4^+^ T cells express NKG2D and be indeed expanded in such disease; a prelude to their potential functional relevance.

**Figure 1:**
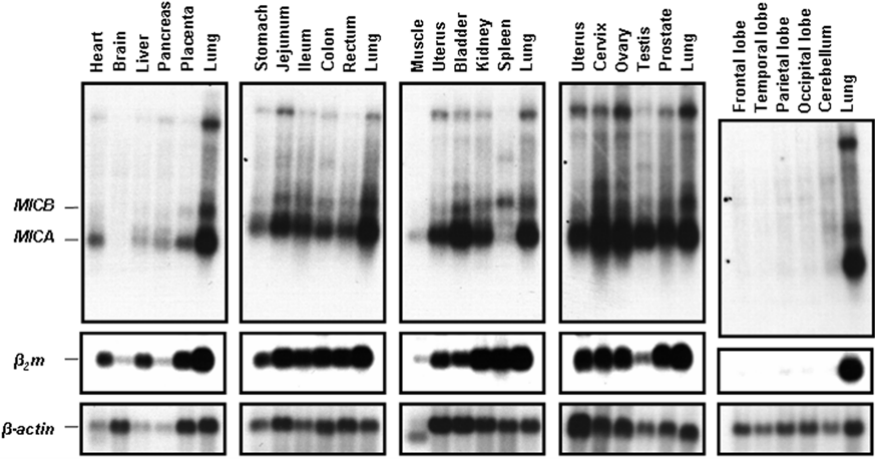
Northern blot analysis of *MICA* and *MICB* expression in various healthy organs. The panel represents RNA originating from major human organs. Each panel contains lung RNA as an internal standard. Top panel was hybridized with *MICA* cDNA. The differential transcript length of *MICA* and *MICB* is due to a previously documented larger 3′UT in *MICB* mRNA [50]. The middle panel depicts the expression of *β_2_m*, indicative of the β_2_m bound conventional MHC-I expression. Finally the *β-actin* transcription acts as a loading control. For more detailed explanation see main text.

**Figure 2:**
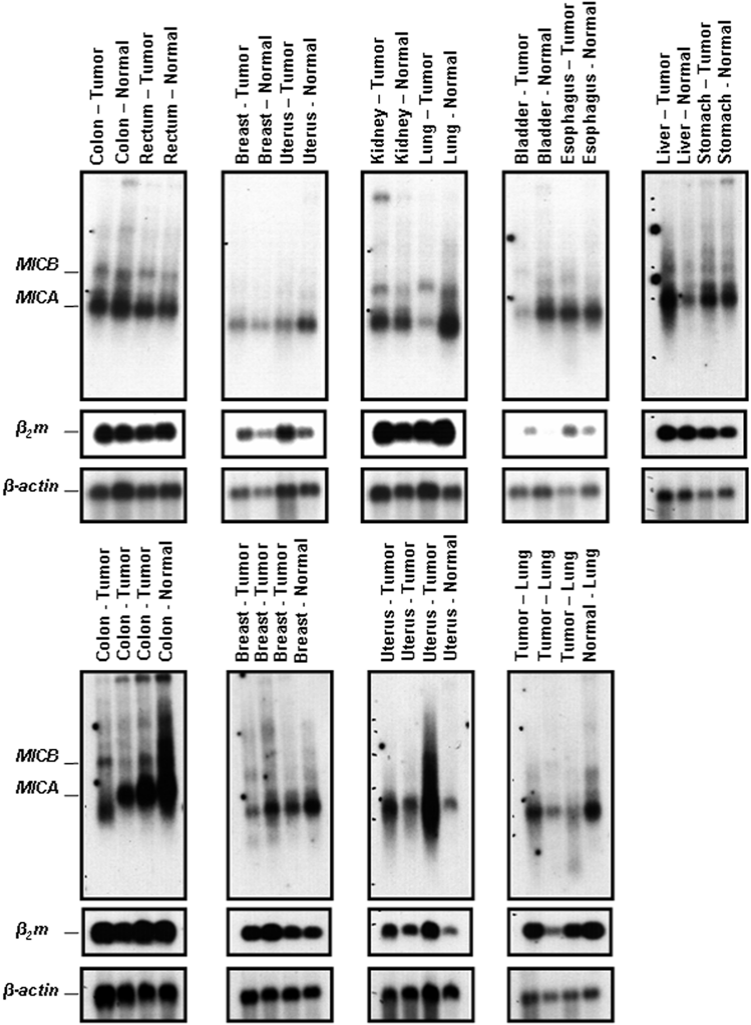
Northern blot analysis of *MICA* and *MICB* expression in various tumors. The same disposition as Figure 1 applies, but this time the blots contain tumor and control (adjacent disease-free tissue) lanes.

Again and although the textbook definition of MIC expression is that of quasi-restriction to enterocytes [20], [24], the reality is far from being so clearcut and this reductionist view simply does not reflect reality (cf. below). Indeed the first monoclonal antibodies raised against MICA proved to stain preferentially, if not quasi-exclusively, human enterocytes, with the notable exception of thymic epithelium [24]. This was in line with initial Northern blottings highlighting the transcription of the gene, not in cell lines (transformed) of lymphohematopoeitic lineage, but in those of epithelial/fibroblastic origin [7]. On the other hand, gene transfer experiments have clearly shown, over and over, that no matter the lineage of the recipient cell line, if the transgene (under a heterelogous promoter that is) is properly transcribed, the MIC protein is invariably synthesized and reaches cell surface [24], [32]–[37], hence excluding any cellular “restriction element(s)” such as those precedently shown to be required for the proper surface expression of other non-classical MHC class I genes e.g. classical HLA signal sequence derived peptides in case of HLA-E/Qa-1 in man/mouse [38] or bacterially-derived N-formylated peptides in case of H2-M3 in mouse [39]. Hence the critical question is in which tissue/organ *MICA* and *MICB* are indeed transcribed, in which case one could confidently presume their consequent surface expression. As trivial as this question might seem, almost 15 years since their identification [7], no systematic transcriptional analysis of *MIC* genes has been published to date. Here we present one first such analysis. Indeed extensive Northern blotting of a large number of human tissues and organs clearly indicates that in contrast to previous contentions, both *MICA* and *MICB* are widely transcribed. As seen in Figure 1 (top panel), these transcripts are found in virtually every organ examined with the notable exception of the central nervous system (top, extreme right panel, Figure 1). This indeed correlates perfectly with the transcription pattern of classical MHC-I loci, as evidenced by probing the same membranes with a *β_2_m* cDNA probe (intermediate panels, Figure 1). Paying closer attention to *MIC* expression pattern, one observes that even an organ “filled” with lymphohematopoeitic cells - the spleen - is not devoid of *MICA* transcripts and contains *MICB* transcripts in equal amounts as to other organs (lung, kidney…). Again, the clear absence of *MIC* from the central nervous system is remarkable, and indeed unexpected, in that it clearly parallels that of classical MHC-I genes (*β_2_m* panel) and this despite the fact that at least the immediate promoter regions of these loci (*MIC* and conventional *MHC-I*) do not show any evident sequence homology [24]. Finally and in order to cover the grounds as much as possible with regards to *MIC* transcription, we extended this transcriptional charting from healthy to tumorized tissues. A number of tumours of various tissue types were analyzed and compared to healthy adjacent tissues (Figure 2). Again, *MIC* transcription was widely present, and no general pattern of induction/repression could be observed in tumours vs. healthy tissues (Figure 2), unlike precedently suggested [13]. Hence, all in all, *MIC* is transcribed in virtually every examined tissue with the notable exception of the central nervous system. The overall pattern in tumours is indicative of a “loose” transcription pattern. The reason why some anti-MIC antibodies have apparently shown to depict a restricted tissue expression pattern must therefore lie elsewhere e.g. genetic polymorphism [40], various N-linked glycosylation patterns (MICA has 8 potential N-linked glycosylation sites) [7], [24].

Having established that *MIC* is transcribed and therefore potentially translated in any/every tissue, we set now to examine the relevance of *MIC* and indirectly that of MIC-NKG2D expression in auto-immunity. For such, three prototypical auto-immune diseases were selected, because of their inherent relevance in understanding human auto-immunity [25], [41], [42], clinical/medical interest as well as the fact that with respect to RA, previous data had pinpointed MIC-CD4^+^NKG2D^+^ interaction in disease aetiology/pathogenesis [15].

**Figure 3:**
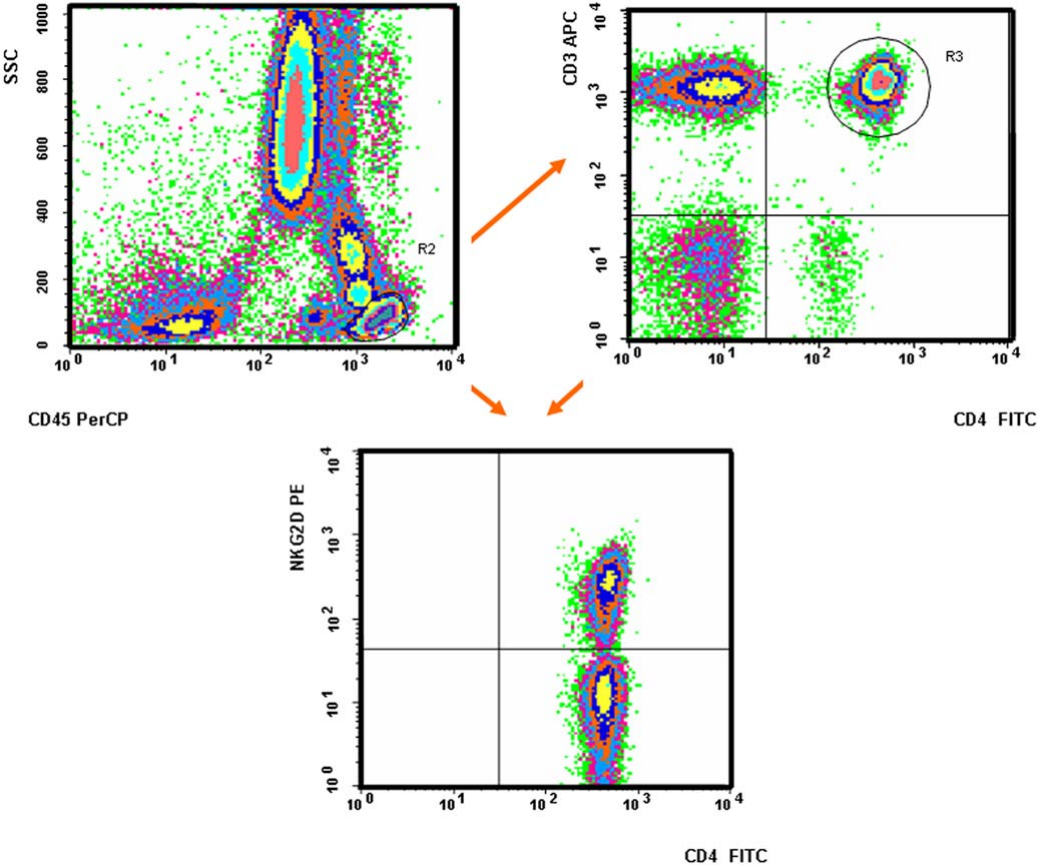
Flow cytometric analysis of the CD4^+^NKG2D^+^ population. This example, taken from one of the two SS patients (Mrs XET) harbouring a particularly high ratio of CD4^+^NKG2D^+^ T cells (36.2%) illustrates the methodology used in order to enumerate these cells in all individuals (controls and patients) included in this work during a routine 50 000 events analysis.

**Figure 4:**
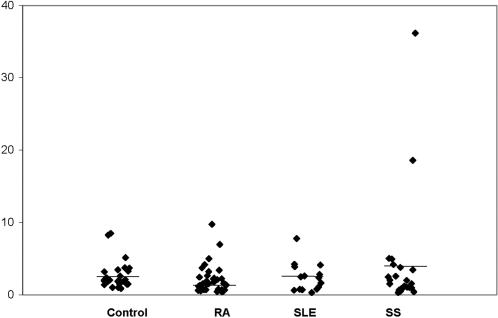
CD4^+^NKG2D^+^ T cells autoimmune diseases. This figure depicts the ratio of CD4^+^NKG2D^+^ T cells within the total peripheral CD4 pool in control, RA, SLE and SS individuals. The variability in SS patients is due to 2 individuals whom represent an unusually high ratio (see main text for explanation). None of the differences is significant as assessed by the Wilcoxon text: p = 0.1658 for controls vs. RA; p = 0.9547 for controls vs. SLE and p = 0.5012 for controls vs. SS.

**Figure 5:**
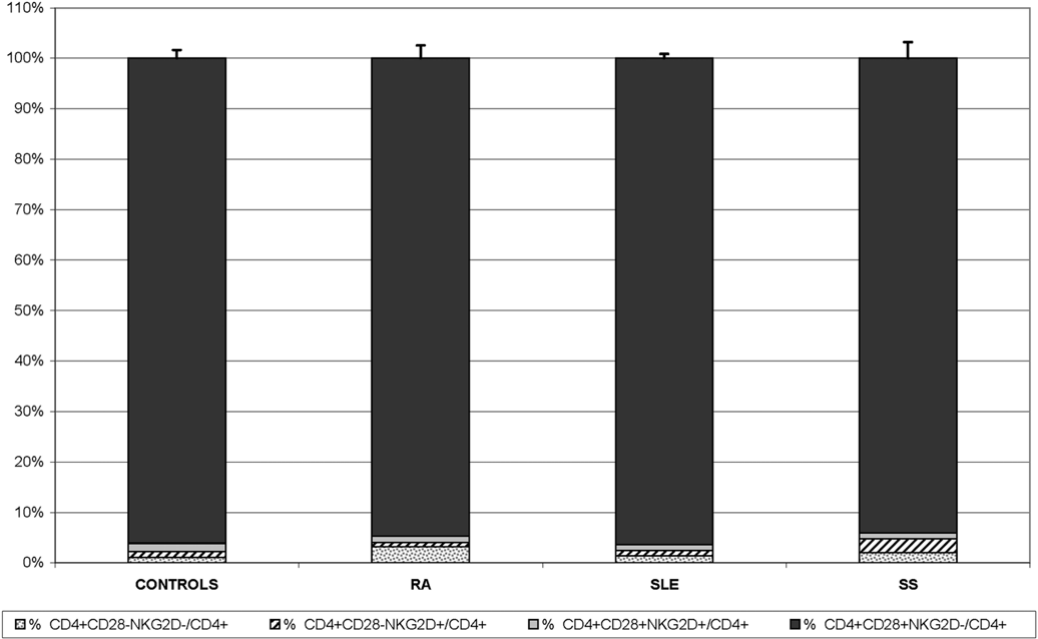
Surface phenotype of CD4 T cells with respect to NKG2D and CD28. This, more global figure, analyses the ratio of CD4^+^ T cells harbouring or not CD28 and/or NKG2D, or not. None of the differences is statistically significant.

For this analysis the following cohort of individuals were gathered: 25 healthy controls and 75 patients suffering from the above mentioned auto-immune disorders i.e. 35 RA, 15 SLE and 25 SS (Table 1). Among these a subgroup including 10 control individuals, 10 RA, 2 SS and 2 SLE patients were extensively immunophenotyped with a wide panel of antibodies, in various combinations, in order to gauge internal standards prior to full scale effort (see Materials and Methods for the full list of antibodies). In this way various T, B and NK cell populations were investigated. No significant difference was observed between the patient and the control populations (data not shown). More specifically the distribution of NKG2D at the surface of αβ CD8^+^ and γδ T lymphocytes as well as NK cells, initially tested using total peripheral blood cell preparations, did not reveal, as expected, any notable difference between patients and controls. We then turned our focus to the population of interest i.e. CD4^+^NKG2D^+^ T cells (Figure 3). The ratio of NKG2D expression on CD4^+^ T cells was found to be equally similar in all healthy volunteers (0.8–8.5%, mean 2.7%, standard deviation 2), all RA (0.4–9.7%, mean 2.1%, standard deviation 1.9), all SLE (0.3–7.8%, mean 2.4%, standard deviation 2) and all SS patients (0–36.2%, mean 4%, standard deviation 7.6) (Figure 4). Two SS patients were indeed found to have higher NKG2D expression on their CD4^+^ T cells (18.6% and 36.2% respectively) (see below for further analysis) (Figures 4 and 3. *NB*: Figure 3 depicts indeed one such patient). Because a precedent study, which had found significantly higher CD4^+^NKG2D^+^ T cells in RA vs. controls, had used another anti-NKG2D mAb, i.e. 1D11 (BD PharMingen) (vs. the above used ON72 clone, Beckman Coulter), we also tested this antibody. Comparison of NKG2D staining with either antibodies on CD4^+^ T cells in 3 healthy volunteers, 4 RA, 1 SLE and 2 SS did not reveal any significant difference (mean difference : 0.21; standard deviation: 0.24). Furthermore we also compared this immunophenotyping comparing total peripheral blood vs. mononuclear cells, using ON72 mAb. This was assessed in 1 healthy volunteer, 2 RA and 2 SS without hinting to any difference (mean difference: 0.33; standard deviation: 0.52). Finally and in order to fully validate the setup, intra-individual stability of NKG2D expression on CD4^+^ T lymphocytes was verified over time in 2 healthy volunteers at respectively days 1/14 and 1/120, for 1 RA patient at d1/d120 and for 2 SS patients at d1/d150. No notable differences (mean: 0.31; standard deviation: 0.5) were observed in different lymphocyte subpopulations and especially those CD4^+^ T cells expressing NKG2D. Given that the same above mentioned report had hinted to the induction of NKG2D expression on CD4^+^ T cells upon adjunction of TNFα (*in vitro*), RA patients under anti-TNFα therapy were not included in this study, despite the fact that a preliminary analysis of three RA patients treated with infliximab did not show any significant difference upon the level of NKG2D expression on CD4^+^ T lymphocytes (data not shown). Hence all in all, we were not able to find any significant difference between the CD4^+^NKGD^+^ population in healthy controls vs. RA, SS or SLE patients. Finally, Figure 5 gives a complete picture of the CD4^+^ T cell pool with regards to NKG2D and/or presence or absence of CD28.

**Figure 6:**
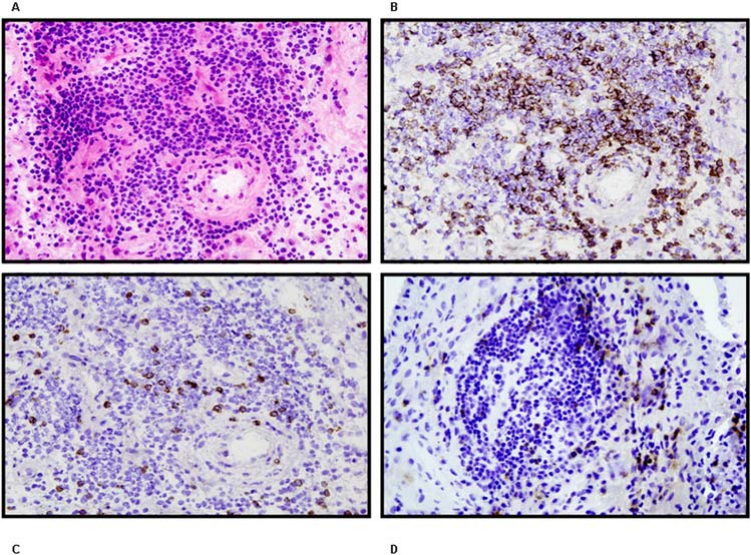
Immunohistochemistry of RA joint infiltrate. (A) Color stain by Hematoxylin-eosin staining. Immunohistochemical analysis using (B) CD4 (C) CD8 and (D) NKG2D antibodies. The infiltrate is clearly of lymphocytic origin with a majority of CD4 T cells.

**Figure 7:**
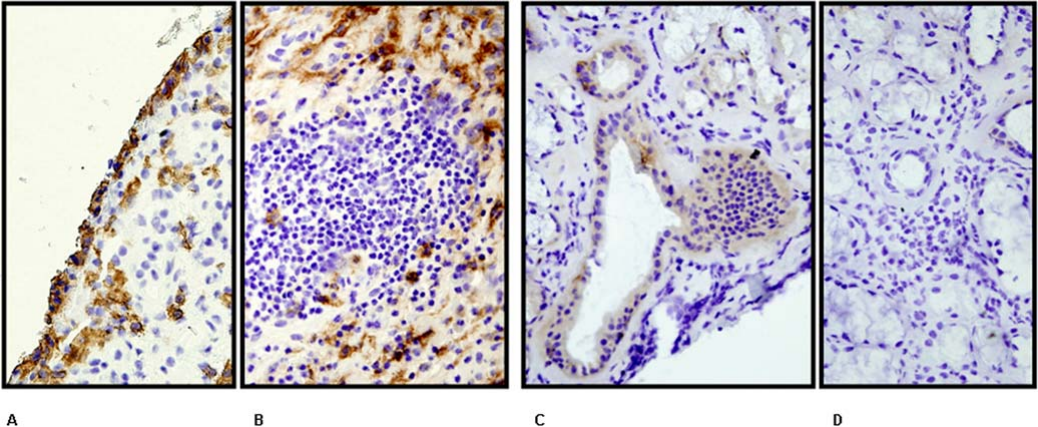
MICA expression in Rheumatoid Arthritis synovitis and Sjögren syndrome accessory salivary glands. Immunohistochemical analysis of (A) (B) RA synovitis and an (C) (D) SS accessory salivary gland stained with an anti-MICA monoclonal antibody. MIC expression is clearly of epithelial/fibroblastic nature in both tissues/organs.

The end-target in RA being the joint, we set out next to analyze the CD4^+^NKG2D^+^ expression levels within mononuclear cells in the articular fluid harvested after Ficoll density gradient centrifugation from one RA elbow and 5 controls (three knee osteoarthritis and two non auto-immune knee arthritis) patients. Again no significant difference in the expression of NKG2D on CD4^+^ T cells was highlighted between controls and RA in joint fluids, and between peripheral blood and joint fluid in one RA patient (data not shown). To further explore the significance of the expression of NKG2D on CD4^+^ and CD8^+^ T cells *in situ*, synovial tissues were analyzed by immunohistochemistry in order to quantify the presence of CD4, NKG2D, CD8 and MICA/B expressing cells. This was performed in one RA (hip arthroplasty) and one osteoarthritis control (knee surgery) individual. In the control individual NKG2D was found to be expressed on 3.8% of peripheral blood CD4^+^ T lymphocytes vs. 2.5% of those found in the joint fluid. As this individual did not carry any active intra-joint inflammation, it was not surprising not to observe a notable infiltration of CD4^+^, CD8^+^ +/− NKG2D^+^ lymphocytes (no MIC expression was found either in this patient). In the RA patient, NKG2D was expressed on 0.9% of circulating CD4^+^ T cells (no joint fluid could be retrieved from the RA hip undergoing surgery). The tissue infiltrate in RA (Figure 6A) was mainly CD4^+^ (Figure 6B) whereas CD8 expression was less important (Figure 6C). NKG2D expression was clearly exclusive of CD4 and more in line with that of CD8 (Figure 6D) (NB.: these are distinct tissue sections, hence no superimposition is meaningful). Finally, MICA/B was expressed on fibroblastic/epithelial cells and not on inflammatory infiltrates (Figure 7A and B).

Although our results in RA are at odds with some of the previously published data, some clarification of the latter might help put our results and indeed the whole question in perspective. Prior to doing such, we clearly do not replicate the work by Groh et al. with respect to expansion of CD4^+^NKG2D^+^ in RA although we did our best to stick to their protocol e.g. using the same anti-NKG2D antibodies etc. [15]. There might be rational explanations for such, one being lack of any detailed information with respect to their patient population, for instance the fraction under anti-TNFα therapy etc. A deeper analysis of the literature indeed shows that the expansion of this time, CD4^+^CD28^−^ T cells, is not as pathognomonic of RA as it might have seemed at the onset [18]. Indeed a more recent analysis of an independent cohort of patients clearly shows that half of the patients with RA (n = 94) (according to authors those devoid of nodular RA and sicca syndrome) did not have a significant increase in their peripheral CD4^+^CD28^−^ T cell pool with respect to controls (see Figure 1 in [43]. Finally Saez-Borderias et al. bring in a new twist to the issue, by reporting the induction of CD4^+^NKG2D^+^ T cells in response to human cytomegalovirus (HCMV), leading to envision the fact that the previous reported expansions may need to be re-assessed in the light of the HCMV serological status of the patients and controls. This also opens the avenue for this subpopulation to recognize, beyond HCMV, other microbial agents, a point not tested in previous (and present) studies [44].

**Figure 8:**
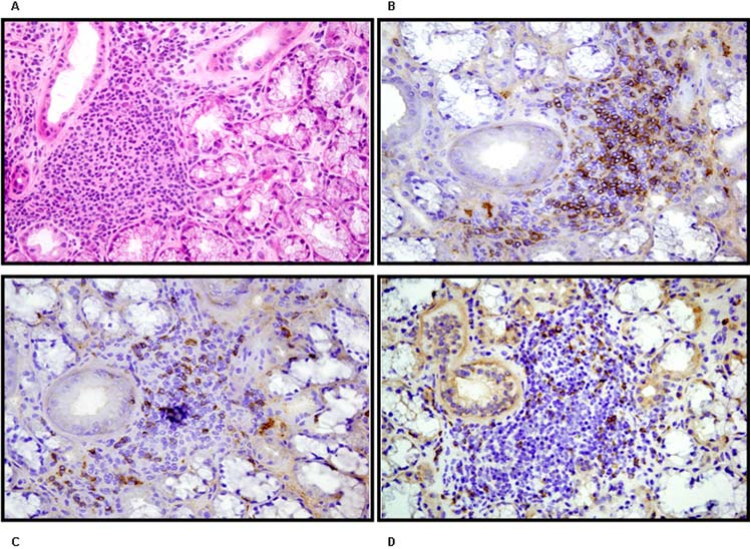
Immunohistochemistry of an accessory salivary gland in Sjögren syndrome. (A) Color stain by Hematoxylin-eosin staining. Immunohistochemical analysis using (B) CD4 (C) CD8 and (D) NKG2D antibodies. The infiltrate is clearly of lymphocytic origin with a majority of CD4 T cells.

To probe the potential significance of CD4^+^NKG2D^+^ T cells in the immunopathology of SS, immunohistochemistry was performed on salivary gland tissues aiming to detect CD4, NKG2D, CD8 and MICA/B. Three SS salivary gland tissues were obtained by biopsy during hospitalization whereas one control salivary gland tissue was obtained from an otherwise healthy volunteer individual during rhinoplasty for independent reasons. In the control individual, the immunohistochemistry did not reveal the presence of any notable numbers of CD4, CD8 and/or NKG2D expressing cells in accord with the absence of any inflammatory infiltrate; this was equally the case for MICA/B expression (data not shown). In SS, NKG2D was expressed on respectively 0.4, 0.9, 2% of peripheral blood CD4^+^ T lymphocytes. Immunohistochemistry (Figure 8A), focalized on inflammatory infiltrates, showed that in all 3 patients, CD4 T cells were most prevalent, again mutually exclusive with CD8 staining (Figure 8B and C). The amount and repartition of NKG2D expression seemed to be comparable to that of CD8 but not that of CD4 (Figure 8D, C and B). Moreover, MICA/B expression was detected on epithelial cells of glandular channels but not on lymphoid infiltrates (Figure 7C and D).

**Figure 9:**
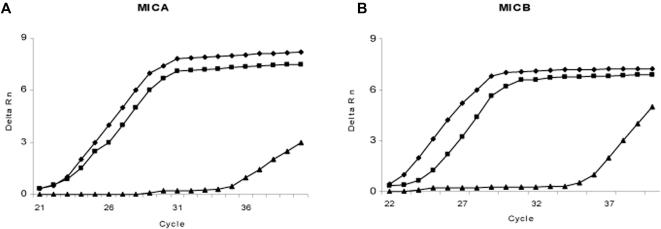
Expression analysis of *MICA* and *MICB* by real-time quantitative PCR in HeLa cells. *MICA* (A) and *MICB* (B) expression profile as analyzed by RT-qPCR. ▪ Samples 15 minutes after heat shock, ♦ Samples 60 minutes after heat shock, ▴ untreated controls.

**Figure 10:**
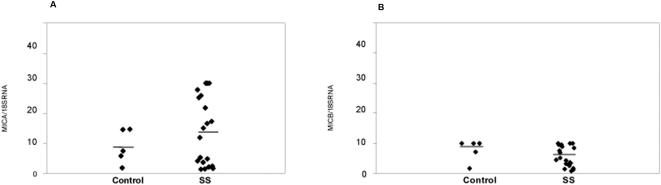
Expression analysis of *MICA* and *MICB* by real-time quantitative PCR in Sjögren's syndrome accessory salivary glands. Relative expression level of *MICA* (A) and *MICB* (B) in Sjögren's syndrome patients as compared to healthy controls. The differences between patients and controls are not significant as assessed by Mann-Whitney *U* test (α = 0.27 for *MICA* and α = 0.53 for *MICB*).

With regards to SS, given the documented expression of MIC genes and proteins in epithelial cells [24], putative involvement of different viruses in SS pathophysiology [45] and the relative ease to access the target tissue, i.e. salivary glands; we aimed to quantify the level of *MIC* transcription in healthy and SS afflicted salivary glands. This was achieved by real-time quantitative PCR analysis of *MICA* and *MICB* transcripts. Prior to doing such on gland RNA, the methodology was validated on HeLa cells; documented to be harbouring *MICA/MICB* transcripts at significant levels, where these are regulated by cell stress e.g. heat shock [7], [24]. Heat shock was performed as previously described [24]. With regards to *MICA* there was an 8x induction after 15 minutes and 3.5× for *MICB*. After 60 minutes the induction remained at 4.5× for *MICA* and 2× for *MICB*. Typical qRT-PCR plots are shown in Figure 9A and B. Within salivary gland tissues, although the expression shows a clear inter-individual variability, no significant difference could be observed between patients and controls (Figure 10A and B).

As alluded to above, among all 75 patients analysed, two SS patients (Mrs. XET and XRL; in this order in this paragraph) did indeed present a particularly high rate of CD4^+^NKG2D^+^ T lymphocytes in peripheral blood (respectively 36.2% and 18.6%). No correlation with age, disease duration, disease activity or treatment was found, and at this stage we consider this phenomenon as fortuitous. These two patients provided nevertheless material to do an extended phenotyping on this peculiar T cell population: 97% and 94% of these CD4^+^NKG2D^+^ T lymphocytes lost CD28, whereas 42.5% and 67.3% expressed CD45RA. Moreover they were negative for CD158a (0.2% and 0.4%), CD158b (0.8% and 0.8%), NKG2A (1.1% and 1.3%) and CD94 (9.1% and 1.3%). The TCR repertoire was analyzed by flow cytometry using 24 V_β_ sub-types. For Mrs. XET, regarding the CD4^+^NKG2D^+^ cells, three V_β_ sub-types were expanded and represented the majority of V_β_ subtypes (V_β_ 13.2 : 19.1%, V_β_ 5.2 : 16.6%, V_β_ 21.3 : 15.4%). For Mrs. XRL, 69% CD4^+^NKG2D^+^ T lymphocytes expressed V_β_ 7.1. So, CD4^+^NKG2D^+^ T lymphocytes expansion seems to have an oligoclonal or monoclonal repartition, the significance of which at this point remains to be established (cf. *infra*: conclusion) (by immunohistochemistry, NKG2D expression on Mrs. XRL salivary gland tissue was not increased and was superimposable to CD8 expression but not to CD4's - data not shown). In the control group, one individual (Mrs Q) with an above baseline CD4^+^NKG2D^+^ T cell count of 8.5% was also further analyzed. 91% of these cells had indeed lost CD28 expression. The repertoire of these cells was biclonal - 41% V_β_ 13.1 and 17% V_β_ 13.6 - in contrast to that of CD4^+^NKG2D^−^ within this same individual (as well as the 2 patients) which did not deviate from the normal range as provided by the V_β_ panel manufacturer. Therefore it seems clear that while CD4^+^NKG2D^+^ T cells are devoid of CD28 (in line with previous findings) this is irrespective of the disease status. This is equally the case for their mono/oligoclonal repertoire.

In conclusion, the sum of this *a priori* diverse set of information presented here may help to set the record straight as to the following: (1) In contrast to what was/is originally/presently assumed and widely written, *MIC* transcription (and for reasons argumented above, translation and surface expression) is not exclusive of enterocytes as virtually every tissue, organ (exception of CNS) does harbour *MIC* transcripts [24], [46]–[49]. It could very well be that within each organ, the transcripts are solely contributed by epithelial and/or endothelial cells, but that would still be in starch contrast to an enterocyte specific expression pattern, not withstanding the case of spleen (cf. supra). (2) The analysis of the same transcription pattern in various tumors helped also nuance the “black and white” message that *MIC* is specifically upregulated in tumors - which is clearly not the case [13]. (3) Finally, it seems clear that CD4^+^NKG2D^+^ T cells - mostly CD28^− ^- are not more prevalent in RA, SLE or SS than in disease-free control individuals. The expansion of these cells in certain individuals, irrespective of health/auto-immune disease, might be due to their recognition of HCMV and/or other infectious agents possibly explaining their mono/oligoclonal T cell repertoire.
